# Dilated Recurrent Neural Networks for Glucose Forecasting in Type 1 Diabetes

**DOI:** 10.1007/s41666-020-00068-2

**Published:** 2020-04-12

**Authors:** Taiyu Zhu, Kezhi Li, Jianwei Chen, Pau Herrero, Pantelis Georgiou

**Affiliations:** Department of Electronic and Electrical Engineering, Imperial College London, London, SW7 2AZ UK

**Keywords:** Dilated recurrent neural network, Diabetes, Continuous glucose monitor (CGM), Glucose forecasting, Deep learning

## Abstract

Diabetes is a chronic disease affecting 415 million people worldwide. People with type 1 diabetes mellitus (T1DM) need to self-administer insulin to maintain blood glucose (BG) levels in a normal range, which is usually a very challenging task. Developing a reliable glucose forecasting model would have a profound impact on diabetes management, since it could provide predictive glucose alarms or insulin suspension at low-glucose for hypoglycemia minimisation. Recently, deep learning has shown great potential in healthcare and medical research for diagnosis, forecasting and decision-making. In this work, we introduce a deep learning model based on a dilated recurrent neural network (DRNN) to provide 30-min forecasts of future glucose levels. Using dilation, the DRNN model gains a much larger receptive field in terms of neurons aiming at capturing long-term dependencies. A transfer learning technique is also applied to make use of the data from multiple subjects. The proposed approach outperforms existing glucose forecasting algorithms, including autoregressive models (ARX), support vector regression (SVR) and conventional neural networks for predicting glucose (NNPG) (e.g. RMSE = NNPG, 22.9 mg/dL; SVR, 21.7 mg/dL; ARX, 20.1 mg/dl; DRNN, 18.9 mg/dL on the OhioT1DM dataset). The results suggest that dilated connections can improve glucose forecasting performance efficiently.

## Introduction

Diabetes is a severe chronic metabolic disorder that causes many secondary complications, such as retinopathy and heart disease. According to the World Health Organization, the global prevalence rate among adults has doubled in the past two decades, reaching 8.5% in 2014 [1]. There are two main types of diabetes: type-1 diabetes and type-2 diabetes. For type-1 diabetes mellitus (T1DM) subjects, their pancreatic β cells lose partial or whole function to produce insulin [2]. There is currently no effective method to prevent T1DM, so people with T1DM require long-term management of blood glucose (BG) to avoid hyperglycaemia (BG above 180 mg/dl) and hypoglycaemia (BG below 70 mg/dl). Such management often requires proper control by means of exogenous insulin delivery, proper diet and exercise.

In general, T1DM subjects need to measure their BG concentration several times per day using standard BG metres. This conventional method normally involves finger-prick tests. Rapid growth of wearable devices for continuous monitoring provide a feasible solution to alleviate this burden. One device is the continuous glucose monitoring (CGM) system, which can continuously measure the BG levels (e.g. every 5 min). Moreover, the CGM, together with the insulin pump, can provide T1DM subjects with closed-loop control systems [3]. These systems can be further enhanced by BG forecasting. The prediction helps the calculation of optimum insulin boluses to avoid possible adverse glycaemic events. Nevertheless, glucose prediction still faces many challenges as there are many daily events that affect BG levels, such as insulin injection, meal intake and exercise. In addition, people with T1DM have large inter-person variability of glycaemic response to insulin, which makes accurate glucose prediction more difficult [4].

Recently, machine learning (ML) techniques have shown great potential for data analysis and prediction. ML approaches focus on learning the behaviours and extracting features automatically from large datasets. There have been several traditional ML applications on diabetes, such as least square support vector machine [5, 6], random forest [7] and *k*-nearest neighbours [8]. As an essential branch of ML, the approaches based on neural networks (NN) have also been implemented in diabetes research. Most of the prior work use artificial neural networks (ANN) with fully connected layers [9–11]. With the ever-increasing computational power and data storage, researchers use a large number of hidden layers to build deep neural networks (DNN). DNN utilises non-linear representations of the layers to extract high-level features and enhance perceptual ability [12]. The superior performance of DNN has been demonstrated in many fields, including medical imaging [13], genetics [14] and using electronic health records (EHR) for the chronic diseases treatment [15].

**Fig. 1:**
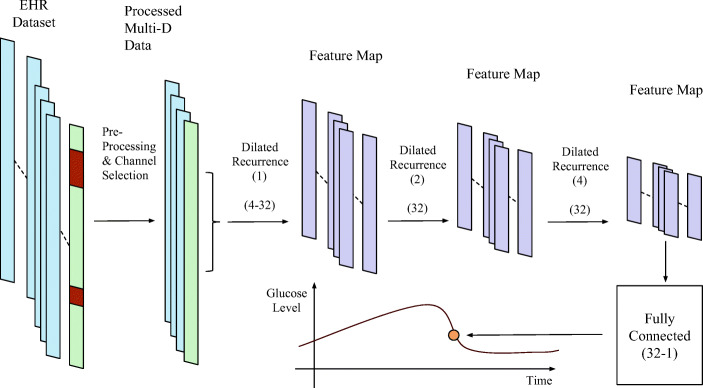
The architecture of the proposed BG forecast model using DRNN layers. There are some missing gaps or outliers, which are shown in orange in the diagram that need to be corrected by pre-processing

In this paper, we propose a deep learning algorithm to forecast glucose concentration for T1DM subjects based on dilated recurrent neural networks (DRNN) [16], as depicted in Fig. 1. The architecture of the DRNN model mainly comprises a series of DRNN layers with different sizes of dilation. Each DRNN layer consists of the recurrent neural network (RNN) cell with plenty of hidden nodes, which can be long short-term memory (LSTM) [17], gated recurrent units (GRU) [18] or simple vanilla cells. The data not only recurrently flows inside the cells to capture the short-term and long-term dependencies but also propagates forward to capture high-level features. The DRNN model is effective to process sequential signals and time series. Moreover, the dilated structure can reduce many parameters and gain higher efficiency [16]. We use Tensorflow to implement the algorithms [19]. We used two EHR datasets to evaluate the performance of the DRNN models: OhioT1DM from clinical trials [20] and *in silicon* dataset from the UVA-Padova simulator [21]. The prediction results of DRNN are compared with many existing algorithms, including neural network for predicting glucose (NNPG) [9], support vector regression (SVR) [22] and the autoregressive model (ARX) [6]. The results show that DRNN achieves the best performance in terms of the root mean squared error (RMSE), the mean absolute relative difference (MARD) and the time lag, which is a common approach to measure the similarity and delay between two time series (prediction and original) using cross-correlation.

## Methods

### Data Acquisition and Pre-processing

We evaluate our models using two datasets from both simulator and clinical trials. To collect the simulated data, we use the UVA/Padova T1D simulator to generate 10 virtual T1DM subjects. It is the only simulator for insulin trials approved by the Food and Drug Administration (FDA) that can provide robust and reliable results [3]. The historical glucose in this dataset is precisely sampled every 5 min and suitable for the model as the input. We generate 103,680 5-min instances for each subject, corresponding to the glucose data samples in 360 days. Each instance contains four data fields: sampling time, CGM values, meal intake and insulin dose. We divide each 360-day dataset into a training dataset and a testing dataset. The clinical OhioT1DM dataset is obtained from six T1DM subjects who wear Medtronic 530G insulin pumps and Medtronic Enlite CGM sensors to provide data during an 8-week period [20]. In addition, there are multiple relative data fields manually reported via an app on a smartphone and a fitness band. The training and testing datasets are provided separately. The total number of instances of each subject in OhioT1DM is different, varying from 13,310 to 15,431. There are 19 data fields for each instance, of which the details are listed in [20]. For the clinical dataset, missing or outlier glucose samples occur frequently. They are due to practical problems, such as the absence of the recording and transmission, errors in measurement and/or mistakes in archiving and documentation. For example, readings from subject 591 illustrates the problem of noisy and missing data; as for a three-day period between the 26 to 29 of December, BG readings for the patient are missing, while prior to this period they remained fixed for a 2-hour period which is unusual and indicates a recording error in the reading.

**Fig. 2:**
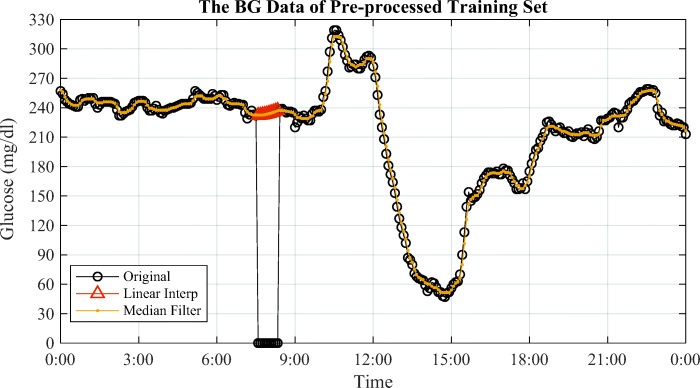
The pre-processed BG training data on Jan 2. Interpolation fills up a small missing interval that the 10 zeros between 7:35 and 8:25, and median filter removes the outliers and spikes on the curve [23]

Consequently, we apply three methods to pre-process the clinical dataset: interpolation or extrapolation, filtering and combination. In particular, we use the first-order interpolation for training datasets to fill up the missing values. Then the training data series pass through a median filter with small window-size to remove spikes and outliers. The data pre-processing of the training set is shown in Fig. 2. Please note that interpolation and median filter are only used for the training. In the testing, extrapolation is adopted because future glucose samples are unknown.

The limited size of dataset possibly degrades the model performance, so we created a generalised training dataset that doubles the length of the original dataset to find out some common features. Specifically, we keep all the data of the current subject accounting for the first half of training set and incorporate data from the other five subjects with 10*%* each to form the second half of the training set. By doing this, we obtain a combined training set. We first train a generalised model on this combined dataset. Then a second phase training is conducted based on the trained model and individual data for a specific T1DM subject by using a transfer learning approach. Experimental results show that this two-phase method increases the size of training dataset and the generality of the model, and it obtains a better prediction accuracy in practice.

### Recurrent Layers

**Fig. 3:**
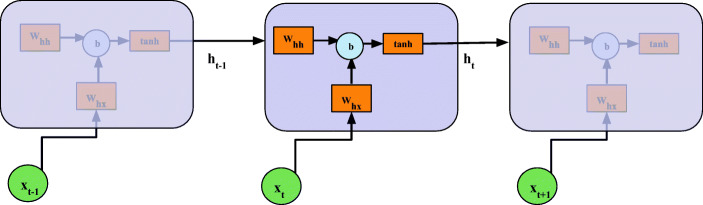
The unrolled structure of vanilla RNN cells

The core backbone of the proposed model is the RNN layers and cells. Compared to conventional NNs, the RNN can remember its input and is powerful at finding patterns of sequential data. It has many applications, such as handwriting generation [24] and machine translation [25]. A sequence of historical multi-dimensional data is fed into the RNN layers as shown in Fig. 1. Basically, each cell contains several hidden states referring to multiple timesteps. The high-dimensional hidden nodes are used in every state to capture feature maps. After tuning a number of hyperparameters as shown in Section 2.5, the vanilla RNN cells are selected in the DRNN architecture because it shows the best overall prediction: the smallest prediction error and the lowest computational expense. Figure 3 depicts the unrolled structure of vanilla RNN layers.

The RNN cell in this architecture derives a conditional probability for the prediction output $p(y_{T}|x_{0},x_{1},\dots ,x_{T})$, where *y*_*T*_ is the prediction target and $x_{0},x_{1},\dots ,x_{T}$ is the input multi-dimensional time series with timesteps of *T*. In the mathematical formulation, the recurrence and the cell output can be expressed as

1 $$ h_{t} = \tanh(\textbf{W}_{hh} h_{t-1}+\textbf{W}_{hx} x_{t} +b) , $$

where *h*_*t*_ is the layer output at timestep *t*,**W**_*hh*_ and **W**_*hx*_ are two weight matrices of hidden states and input, respectively, *b* denotes the bias, *h*_*t*− 1_ is the output from last timestep and *x*_*t*_ stands for the current input vector. Each hidden layer uses distinct weight matrices. It is noted that the parameters of vanilla architecture in (1) are much less than LSTM and GRU (details in Table 1). Thus, the vanilla RNN cells are efficient to train the whole network with less time and computational resource.

**Table 1:** RMSE performance for traditional LSTM and different DRNN models

Model	Traditional LSTM	DRNN (Vanilla)	DRNN (LSTM)	DRNN (GRU)
Simulated dataset	9.2^∗∗^	7.8	7.9	7.9
OhioT1DM dataset	21.0^∗∗^	18.9	20.2^∗^	19.9
Number of parameters	4(*n*^2^ + *m* × *n* + *n*)	*n*^2^ + *m* × *n* + *n*	4(*n*^2^ + *m* × *n* + *n*)	3(*n*^2^ + *m* × *n* + *n*)

^∗^*p* ≤ 0.05^∗∗^*p* ≤ 0.01

### Multi-layer Dilated Connections

**Fig. 4:**
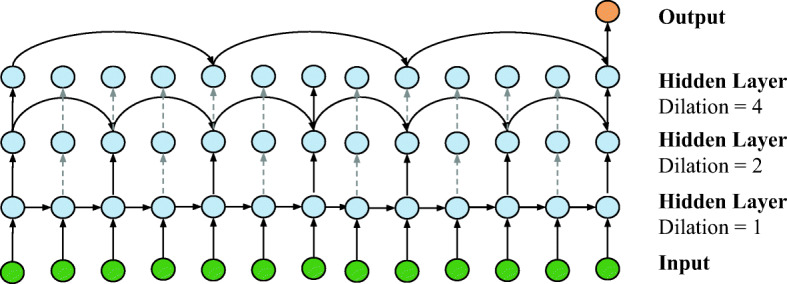
An illustration of dilated connections of multi-layer RNN architecture. The dilation in three layers increases exponentially from 1 to 4

In general, vanilla cells face the problem of gradient vanishing, but the DRNN architecture tackles this challenge and improves the performance [16]. The concept of dilation for RNNs means fetching the previous state output after skipping certain numbers of timesteps in each hierarchical layer. As such, DRNN architecture has a stack of cell-independent layers to realise multi-resolution. This structure makes the vanilla cell outperform LSTM and GRU. Figure 4 presents a detailed illustration of the proposed DRNN model.

We feed a vector as the input dictionary containing data from the multiple EHR fields at each timestep. The bottom DRNN layer has dilation of 1, which is the same as the standard structure of vanilla RNN cells as shown in Fig. 3. For higher hidden layers with dilation *d*, the RNN cells fetch the state input by skipping *d* − 1 timesteps. Due to the flexibility of the RNN, we have a many-to-one structure: one predicted value is obtained by considering a sequence of historical multi-dimensional data. Specifically, the dilated connections with *d* > 1 and the cell output can be formulated as

2 $$ \begin{array}{@{}rcl@{}} {h_{t}^{l}} &= &f(h_{t}^{l-1},h_{t-d^{l}})\\ &=&\tanh(\textbf{W}_{hh} h_{t-d^{l}}+\textbf{W}_{hh^{l-1}} h_{t}^{l-1} +b^{l}), \end{array} $$

where ${h_{t}^{l}}$ denotes the output of layer *l* at timestep of *t* and *f*(⋅) stands for any RNN cell operation, including GRU, LSTM and vanilla RNN. Particularly, the second line of the (2) expands the expression for vanilla cells. After passing through the DRNN layers within total times steps *T*, the final output with *L* DRNN layers is denoted as ${h_{t}^{l}}$. Then the output vector propagates into a fully connected layer to regress toward the scalar predicted values.

As illustrated in Fig. 4, the DRNN uses fewer parameters to compute a single prediction compared to fully connected RNNs. Hence, it enhances the training efficiency and alleviates the gradient vanishing problem. Moreover, the DRNN allows parallel operations that are quite suitable for GPU computation [16]. The cell chains can be computed in parallel using shorter sub-sequences. Taking the second layer with dilation of 2 in Fig. 4 as an example, the computation of the chains of cell outputs *h*_2*k*_,*h*_2*k*+ 2_ and *h*_2*k*+ 1_,*h*_2*k*+ 3_ can be accomplished in parallel. Thus, it further reduces the training time cost with a parallel implementation. Other important benefits of DRNNs are brought by exponentially increasing dilation ([1,2,4] in Fig. 4) [16]. On the one hand, the different dilation guarantees a variety of temporal resolution of time series. On the other hand, it effectively reduces the average path length of recurrence between two timesteps by skipping nodes. Therefore, the DRNN models also have superior capabilities to capture long-term dependencies. It partially explains the better performance of the vanilla cell, because it only focuses on the short-term dependencies with the simplest structure and leaves long-term patterns to dilated cell connections.

### Network Training

After the DRNN model construction, we start training the network to obtain the optimal weights and bias with a large amount of data in batches. In the ML field, we usually use the gradient descent algorithm to update network weights. Loading a large dataset requires hardware with high memory capacity, and it is more likely to end with a local optimum instead of the global optimum. Therefore, in our model, we apply the mini-batch approach by using a subset of the data to update parameters for each training step [26]. During the process of training, the hyperparameters are tuned as described in Section 2.5. Three fields of the pre-processed data are chosen as the input channels: historical BG (G), insulin bolus (I) and meal intake (M). In addition, a time index (T) channel is added by normalising the one-day duration of 288 samples into a range of [0,1). For example, 0:00 and 12:00 refer to 0 and 0.5, respectively. The target label **y** is set to the change between current BG(*x*_*t*_) and future BG (*x*_*t*+ 6_).

**Fig. 5:**
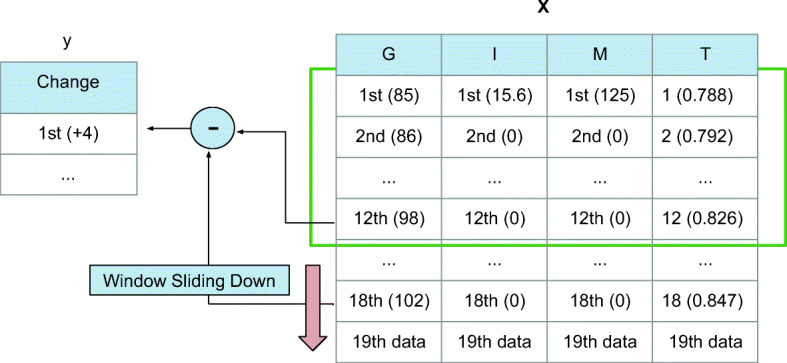
An illustration of making batches with real examples from the clinical dataset. Sliding down the windows, we obtain multiple entries in a batch

Figure 5 shows the process of making batches by sliding down the window step by step to extract a set of sequential data as well as a label. These batches are directly fed into the model. In our model, a conventional method of adaptive learning rate, RMSprop optimiser, is applied [27]. After sending a number of batches to the network, we calculate the error between predicted values and labels, average the loss, and then use backward propagation to update parameters. The outcome at the top fully connected layer is the change value of BG concentration for the PH ahead. We add it to the current BG to obtain the future glucose level. This process can be written as

3 $$ \hat{y}_{T} = {\Delta} BG+x_{T} = \mathbf{W_{FC}}{h^{L}_{T}}+b_{FC}+x_{T},  $$

where $\hat {y}_{T}$ is the future BG prediction, Δ*BG* is the prediction value of the change, *x*_*T*_ denotes current BG, *W*_*FC*_ is the weight matrix and *b*_*FC*_ is the bias. In other words, *W*_*FC*_ and *b*_*FC*_ are applied to the orange output in Fig. 4. We follow the default non-linearity in the DRNN architecture, so there is no activation function in the fully connected layer.

### Hyperparameter Tuning

When constructing DRNN models, there are a number of hyperparameters that need to be tuned manually. The combinatorial space is not very large, so we have performed grid search for each hyperparameter. We extracted 10% data from the end of training datasets as the validation datasets and kept the first 90% data to train the models. Hence, for the simulated dataset, the overall split is 81% for training, 9% for validation and 10% for testing. Considering the task is to predict time series using historical data, we used these validation datasets as 90*%* and 10*%* partition instead of conventional *k*-fold cross-validation. The evaluation process and final hyperparameters in Table 2 are identical for all subjects in simulated and clinical datasets.

**Table 2:** DRNN hyperparameters

Parameter	Value
Length of sequences	12 timesteps
Input channels	[G, I, M, T]
Cell type	Vanilla RNN
Dilation	[1, 2, 4]
Hidden node dimension in each layer	32
RMSprop learning rate	0.001
RMSprop decay rate	0.9
Batch size	512

We feed DRNN with a sequence containing historical data. The length of sequences was tuned among {6, 12, 18}, corresponding to the data in previous {30, 60, 90} min. Using the RMSE in (4) as the indicator of L2-loss, we have observed that the model has the smallest RMSE when length equals to 12. From the datasets of T1DM subjects, we incorporated historical BG (G), insulin bolus (I), meal intake (M) and time index (T) and sent them to the DRNN as separate channels. The historical G channel is necessary for prediction, so we explored channel combinations among {G, [G, I], [G, M], [G, I, M], [G, I, M, T]}. The results show that the input with four channels of [G, I, M, T] performs the best.

Table 1 presents the mean RMSE results for different RNN models. We constructed the traditional LSTM model by setting the dilation to [1, 1, 1] and compared DRNN models among cells of {Vanilla,LSTM,GRU}. Assuming a cell state has an *n*-dimensional hidden node and an *m*-dimensional input, the number of parameters in each state is shown in Table 1, according to [28]. It is noted that, compared with vanilla RNN, the LSTM and GRU architecture have a 4-fold and a 3-fold increase in parameters, respectively. Considering DRNN (LSTM) and traditional LSTM, we can see that the dilation helps to reduce the RMSE and improves the performance. Among DRNN models, we observed the vanilla cells have the best RMSE. Although the RMSE of vanilla cells and GRU are close, especially on simulated datasets, we still selected the vanilla RNN, considering its low computational expense as well as a simple structure as shown in Fig. 3.

For the dilation, we first followed the settings in [16] and used five layers with dilation of [1, 2, 4, 8, 16], but we observed overfitting occurred when validation loss was significantly larger than training loss. We then reduced the complexity of the model and found that three layers with dilation of [1, 2, 4] performed best. We tuned the hidden node dimension among {16, 32, 64, 128} and mini-batch size among {256, 512, 1024} in a similar process, by observing the training and validation performance. For RMSprop optimiser, we selected the learning rate of 0.001, comparing the values of {0.01, 0.001, 0.0001}, and a value of 0.9 for the decay rate.

## Results

In this section, we compare the DRNN model with three prediction algorithms in recent works: NNPG, SVR and ARX. Using grid search, we carefully tuned the hyperparameters for each algorithm to build a fair comparison. For the SVR model, we first explored the kernels among RBF, Linear, Polynomial and Sigmoid. Then the coefficient of *γ* was tuned among for RBF, Polynomial and Sigmoid, in a range of [0.001, 10]. The penalty term *C* was tuned between [0.1, 1000]. Based on the tuning performance, SVR is developed in Python environment with RBF kernel and settings (*C* = 100,*γ* = 0.01,*cache*_*size*_ = 1000). NNPG is an application of ANNs with three fully connected layers, realised by Keras in Python. For NNPG’s hyperparameters, the search space for learning rate is [0.0001, 0.1], and batch size is tuned in the range of {8, 16, 32, 64}. The learning rate of 0.001 and batch size of 32 are finally selected, respectively. For the ARX algorithm, we use the function *arx*() with the 3^rd^ order by the library of MATLAB. The input batch of testing sets is the same as training sets in Fig. 5, including historical BG (G), insulin bolus (I), meal intake (M) and time index (T). To assess the significance between the DRNN models and other considered methods, the *t* test is applied for calculating *P* values, where *P* < 0.05 and *P* < 0.01 stand for statistically significant. The PH in this work is set as 30 min because it is widely used in existing work [6, 9, 22]. Besides, this PH is also suitable for T1DM subjects to take actions in advance to prevent adverse glycaemic events [29].

### Criteria for Assessment

There are three criteria applied to evaluate the performance of testing, since a single metric is not sufficient to provide a comprehensive analysis. In particular, we use the RMSE and the MARD as the principal indicators to measure the error between prediction BG levels and original values, and the time lag to measure the delay of the prediction. The formulation of the RMSE is expressed as

4 $$ { \textbf{RMSE}} = \sqrt{{1\over N}\sum\limits_{t=1}^{N}(\hat{y}_{(t|t-PH)}-y_{t})^{2}},  $$

where *N* denotes the total number of BG points in the dataset, $\hat {y}_{(t|t-PH)}$ is the prediction of **x**_*t*−*PH*_ and *y*_*t*_ is the original value. Similarly, the MARD is given by

5 $$ { \textbf{MARD}} = {1\over N}\sum\limits_{t=1}^{N}|(\hat{y}_{(t|t-PH)}-y_{t})/y_{t}| \times 100<percent>.  $$

With the cross-correlation of the predicted and actual BG values, the time lag *τ*_*lag*_ is formulated as

6 $$ \tau_{lag} = \underset{k}{\arg\max} (\hat{y}_{k}(k|k-PH) \star {y}(k)). $$

Using RMSE and MARD to evaluate the glucose prediction is a common approach in many previous works [6, 9, 22, 30]. RMSE can effectively present the overall performance of the prediction models. MARD reflects the relative error to the current glucose levels and alters the risk of hypoglycemic [31]. The time lag evaluates how fast the prediction models react to the abrupt changes of BG levels [9, 30].

### Performance on the Simulated Datasets

Using UVA/Padova T1D simulator, we generate 10 adult subjects with a length of 360 days. The meal intake is set to 3 times per day without fixed schedule. We vary the times of insulin events between 1 to 5, assuming they are not necessary to be taken with the meal. The intra-day variability for meal size and time is set to *CV* = 10*%* and *STD* = 20, respectively [32]. Exercise is also considered as a daily event and set to *CV* = 10*%*, although it is not used as an input channel. The intra-subject variability is applied to guarantee the uniqueness of each subject case. We use the data in the first 324 days to train the model and save the rest data of 36 days as the testing datasets. The PH is set to 30 min for all the algorithms.

Table 3 presents the average RMSE and MARD results of 10 adult cases to compare prediction methods. Notably, the DRNN model performs best with the smallest mean RMSE and MARD. The DRNN model achieves mean RMSE of 7.8 mg/dl that is much lower than others best (ARX, 11.3 mg/dl). This is a remarkable improvement in forecasting BG concentration. Meanwhile, the DRNN model has the RMSE and MARD results with the smallest standard deviation, which accounts for its generalised optimisation to various individual subjects.

**Table 3:** Prediction performance for the 10 simulated T1DM subjects

Method	DRNN	NNPG	SVR	ARX
RMSE (mg/dl)	7.8 ± 0.6	13.1 ± 1.2^∗∗^	11.9 ± 1.4^∗∗^	11.3 ± 0.8^∗∗^
MARD (%)	4.8 ± 0.6	7.2 ± 1.1^∗∗^	6.1 ± 0.8^∗∗^	6.8 ± 0.9^∗∗^
*τ*_*lag*_ (mins)	0.4 ± 0.3	9.3 ± 1.8^∗∗^	6.8 ± 1.6^∗∗^	4.8 ± 1.5^∗∗^

^∗^*p* ≤ 0.05^∗∗^*p* ≤ 0.01

**Fig. 6:**
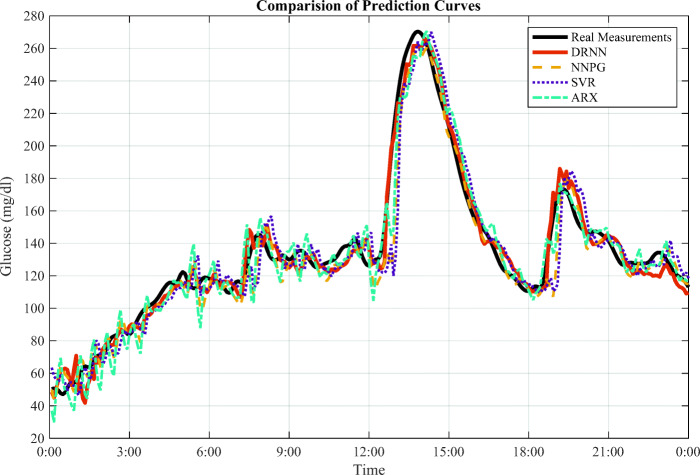
The forecasting curves for virtual adult 1 in one-day period. It is an average scenario from the testing dataset

Figure 6 presents the prediction curves for the simulated case of adult 1. There are three peaks observed around 8:00, 14:00 and 19:00, referring to the meal intakes. Particularly, the DRNN model reacts rapidly near the turning points of peaks, and its curve fits the sharp uptrends and downtrends much better than other methods. This point is supported by the results of *τ*_*lag*_ in Table 3, where DRNN has the shortest time lag. For the regions with the slow change, the DRNN curve also fluctuates gradually and fits them well. These observations from Fig. 6 also accord with the smallest RMSE and MARD performance by the DRNN model.

### Performance on the Clinical Dataset

As mentioned in Section 2.1, the clinical dataset, namely OhioT1DM, has six T1DM subjects indexed as 559, 563, 570, 575, 588 and 591, which is provided by Blood Glucose Level Prediction Challenge in KDH 2018 [20]. The data has already been divided into a training dataset with the length of around 40 days and a testing dataset of around 10 days. The DRNN model ranked top in the challenge, achieving relatively high BG prediction performance [33].

Table 4 shows the specific RMSE and MARD results of six T1DM subjects with four prediction methods. The DRNN model still exhibits the best performance with the smallest RMSE and MARD for each T1DM subject. Overall, compared with the simulated dataset, the RMSE and the MARD both increase, as the prediction performs worse. The ranking of forecasting accuracy (average RMSE) of different methods remains the same as the results of the simulated dataset: DRNN> *ARX* > *SVR* >NNPG.

**Table 4:** Prediction performance for the 6 clinical T1DM subjects

Subject	559	563	570	575	588	591	Avg ± SD
DRNN							
RMSE (mg/dl)	18.6	18.0	15.3	22.7	17.6	21.1	18.9 ± 2.6
MARD (%)	8.6	8.0	5.6	10.3	7.9	11.9	8.7 ± 2.2
*τ*_*lag*_ (mins)	4.8	6.3	2.2	12.3	8.6	14.1	8.1 ± 4.1
NNPG							
RMSE (mg/dl)	23.3	21.2	19.0	27.4	21.9	24.9	22.9 ± 2.9^∗∗^
MARD (%)	10.2	9.4	7.1	13.4	9.7	14.8	10.8 ± 2.8^∗∗^
*τ*_*lag*_ (mins)	12.8	16.4	11.8	21.4	13.6	22.6	16.4 ± 4.2^∗∗^
SVR							
RMSE (mg/dl)	23.5	18.3	20.4	23.6	20.6	23.5	21.7 ± 1.9^∗^
MARD (%)	9.8	8.3	6.2	10.2	8.4	12.6	9.2 ± 2.2^∗^
*τ*_*lag*_ (mins)	11.4	15.8	10.3	20.4	12.3	21.4	15.3 ± 4.3^∗∗^
ARX							
RMSE (mg/dl)	18.7	19.6	16.8	23.6	19.5	22.4	20.1 ± 2.5^∗^
MARD (%)	8.0	8.4	6.0	10.6	8.3	12.0	8.9 ± 4.0
*τ*_*lag*_ (mins)	8.4	13.4	8.6	18.1	11.8	21.3	13.6 ± 4.8^∗∗^

^∗^*p* ≤ 0.05 ^∗∗^*p* ≤ 0.01

**Fig. 7:**
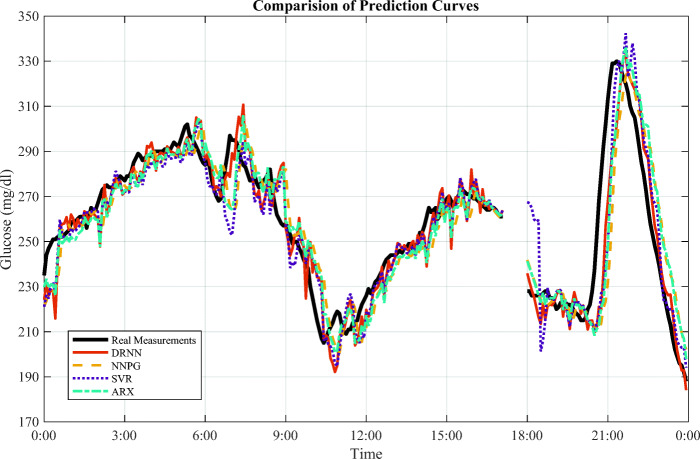
The prediction curves for clinical subject 570 on Jan 21 from the testing dataset. There is a missing interval between 17:10 and 18:00, which contains 10 BG measurements

Among the clinical subjects, 570 has the smallest RMSE of 15.3 mg/dl and MARD of 5.6%. Observing their training and testing data, it is noted that the number of missing data instances is 1309 for subject 575, while subject 570 only has 649 missing points of short intervals. Hence, the quality of the data has an essential influence on prediction performance, although we use multiple steps to pre-process data. In fact, the missing interval appears every day in the dataset, as the 50-min gap in Fig. 7. Thus, we use linear extrapolation to estimate the missing data for the testing set based on the previous trend.

In Fig. 7, we compare the four prediction algorithms by their forecasting curves for subject 570 on January 21. Similarly, the DRNN is quite sensitive. It has a rapid response to the abrupt change of glucose levels, corresponding to the smallest *τ*_*lag*_ in Table 4. The delay becomes particularly large for the uptrends, whereas the DRNN curve fits the downtrends of the peaks quite well. For the nearly linear or slow changing regions, the DRNN can forecast the future BG levels with relatively small error.

## Discussion

### Performance Analysis

Comparing the overall performance using the two datasets, the simulated dataset resulted in smaller RMSE than the clinical dataset. This is because the scenarios experienced by real T1DM subjects are much more complicated than those given in UVA/Padova T1D simulator. There are many practical factors that can influence blood glucose, such as illness and stress. For those factors, the hidden non-linearity makes it difficult for DNN models to learn, although we have introduced various activation functions into the network layers. Another reason for the degraded performance on the clinical dataset is the defects in the dataset, as discussed in Section 2.1. The strategy to address this problem is to use pre-processing. We found that these pre-processing steps can improve average RMSE by 0.3 mg/dl in the experiments. Interpolation and extrapolation improve the performance of each subject, while the transfer learning is especially effective to subject 591 that has a long-missing interval.

An interesting point in Fig. 7 is the DRNN curve fits better at downtrends than uptrends. According to the EHR of subject 570, the rapid increase at 19:00 of BG concentration happened after meal intake, followed by an insulin bolus that reduced glycaemia quickly. Thus, one explanation for this phenomenon is that people manually reported their estimates of meal information (like the time and carbohydrate intake) possibly making them less reliable than the values of insulin dose.

### Limitations and Future Work

Although the DRNN model delivers state-of-art results and exceeds many existing algorithms, there are still some limitations. We develop a purely data-driven model that heavily relies on historical EHR. The quality of the data has a critical role in the prediction performance. However, the size of clinical datasets is often limited. Several data fields are recorded manually, so sometimes they are inaccurate. To improve the data quality, diabetes dynamics values could be integrated with data pre-processing, for instance: *Pi* (plasma insulin estimation) and *Ra* (glucose rate of appearance). In most cases, the DRNN algorithm provides effective prediction for users so people can have glucose intervention in time. However, it has a chance to miss some alerts on potential hypoglycemia if users conduct exercise with high intensity. Further research is required to integrate factors of exercise with the existing algorithm. In addition, we have embedded the presented algorithm into an iOS app which will be evaluated in a clinical trial in the near future.

Meanwhile, we can employ a hybrid algorithm of different approaches in future work. For example, we can use shallow neural networks as the bottom layers, such as fully connected or convolutional layers to extract features and pre-process the data. Then, the feature maps are fed into the DRNN to obtain high-level features of time series, similar to [30]. For state-of-the-art techniques, we consider using the DRNN layer as a basic unit in generative adversarial network and reinforcement learning for diabetes management. It is found that the dilation structure also benefits other types of neural networks in processing time-aligned signals [23, 34]. We will focus on the improvement of the dilated architecture. Moreover, we aim at building a user-friendly deep learning platform for diabetes analysis, so researchers with little knowledge in data science are able to develop DNN models conveniently [35].

## Conclusion

In this paper, a deep learning model to forecast glucose concentration for T1DM subjects is proposed using dilated recurrent neural networks. After data pre-processing, a multi-dimensional time series, including historical blood glucose, insulin bolus, meal intake and time index, is fed to the network to obtain the glucose prediction. The main architecture of this model consists of several dilated recurrent layers and a fully connected output layer. Compared with the standard RNNs, the recurrent layers in the DRNN model exponentially increase dilation to expand their receptive fields and improve the prediction accuracy. With a properly trained model, we conducted an evaluation with a new set of data and compared its performance with the results of the NNPG, SVR and ARX methods. Our results show that the DRNN model achieves the best performance with the smallest RMSE, MARD and time lag. Therefore, we believe the DRNN model is a promising approach to achieve good BG prediction and has great potential for future research in diabetes management.
